# DNase Treatment Improves Viral Enrichment in Agricultural Soil Viromes

**DOI:** 10.1128/mSystems.00614-21

**Published:** 2021-09-07

**Authors:** Jackson W. Sorensen, Laura A. Zinke, Anneliek M. ter Horst, Christian Santos-Medellín, Alena Schroeder, Joanne B. Emerson

**Affiliations:** a Department of Plant Pathology, University of California, Davis, Davis, California, USA; b Genome Center, University of California, Davis, Davis, California, USA; California State University, Northridge

**Keywords:** DNase, relic DNA, viromics, metagenomics, soil, viruses

## Abstract

The small genomes of most viruses make it difficult to fully capture viral diversity in metagenomes dominated by DNA from cellular organisms. Viral size fraction metagenomics (viromics) protocols facilitate the enrichment of viral DNA from environmental samples, and these protocols typically include DNase treatment of the post-0.2-μm-filtered viromic fraction to remove contaminating free DNA prior to virion lysis. However, DNase may also remove desirable viral genomic DNA (e.g., contained in virions compromised due to frozen storage or laboratory processing), suggesting that DNase-untreated viromes might be useful in some cases. In order to understand how virome preparation with and without DNase treatment influences the resultant data, here, we compared 15 soil viromes (7 DNase treated and 8 untreated) from 8 samples collected from agricultural fields prior to tomato planting. DNase-treated viromes yielded significantly more assembled viral contigs, contained significantly less nonviral microbial DNA, and recovered more viral populations (viral operational taxonomic units [vOTUs]) through read mapping. However, DNase-treated and untreated viromes were statistically indistinguishable in terms of ecological patterns across viral communities. Although the results suggest that DNase treatment is preferable where possible, in comparison to previously reported total metagenomes from the same samples, both DNase-treated and untreated viromes were significantly enriched in viral signatures by all metrics compared, including a 225-times-higher proportion of viral reads in untreated viromes compared to total metagenomes. Thus, even without DNase treatment, viromics was preferable to total metagenomics for capturing viral diversity in these soils, suggesting that preparation of DNase-untreated viromes can be worthwhile when DNase treatment is not possible.

**IMPORTANCE** Viromics is becoming an increasingly popular method for characterizing soil viral communities. DNase treatment of the viral size fraction prior to DNA extraction is meant to reduce contaminating free DNA and is a common step within viromics protocols to ensure that sequences are of viral origin. However, some samples may not be amenable to DNase treatment due to viral particles being compromised either in storage (i.e., frozen) or during other sample processing steps. To date, the effect of DNase treatment on the recovery of viruses and downstream ecological interpretations of soil viral communities is not thoroughly understood. This work sheds light on these questions and indicates that while DNase treatment of soil viromes improves the recovery of viral populations, this improvement is modest in comparison to the gains made by viromics over total soil metagenomics. Furthermore, DNase treatment may not be necessary to observe the ecological patterns structuring soil viral communities.

## INTRODUCTION

Viruses infect all three domains of life and play key roles not only in human health but also in agriculture and global nutrient cycling (1–5). They are important in oceanic food webs, and our understanding of their role in soils is growing rapidly (2, 6–14). Viral abundances are estimated to range from 10^7^ to 10^10^ virions per g in soil (6, 11), and measurements from transmission electron microscopy suggest that up to 28% of microbial cells in soil are actively infected by viruses (15–17). Through metagenomic approaches, soil viral populations have been implicated in soil carbon cycling and microbial community dynamics in changing environments, including in thawing permafrost and other peatlands (18–20).

The study of soil viral communities has lagged behind analogous efforts in marine systems, in part because the complex and heterogeneous nature of soil presents unique challenges for recovering viral DNA (2, 10, 11, 14, 21). Although marine viral ecology has benefited from a viromics approach, in which purified, concentrated viral particles are subjected to DNA extraction and metagenomic sequencing (12, 22, 23), most recent soil viral ecological studies have focused on recovering viral signatures from total soil metagenomes (10, 19, 20, 24). Bioinformatic advances in viral contig identification (e.g., through the recognition of viral hallmark genes and other viral sequence signatures) (25–28) and efforts to compile viral reference databases that include partial and putative viral genomes (1, 19, 29) have improved our ability to recognize viral genomic sequences in soil metagenomes. However, despite these advances, our ability to catalog soil viral diversity is still largely gated by the low prevalence of viral DNA in soil and other metagenomes, which tend to be dominated by bacterial and archaeal sequences (10, 19, 30).

Fortunately, viral size fractionation protocols (e.g., the passage of a sample through a 0.2-μm filter to remove most cells), initially used in marine and other aquatic systems (12, 22, 23, 31–34), have also been applied to soil (11, 35, 73), and recent data suggest that these protocols can enrich the viral signal in sequencing data (10, 19, 21, 30). Through iterative steps of mechanical and/or chemical desorption and centrifugation, virus-sized particles are separated from the soil matrix and microbial cells, and DNA can then be directly extracted and sequenced from this viral size fraction to generate a shotgun metagenome, known as a virome (11, 18, 19, 30, 36, 37). Our group has shown that this approach can greatly increase both the number of viral populations and the proportion of viral DNA in the produced sequencing data from soil viromes, compared to total metagenomes (19, 30). For example, in agricultural soils, on average, 30 times more contigs were identified as viral and 585 times more reads were recruited to viral genomes in viromes than in total metagenomes from the same samples (30).

A common step in laboratory viromics protocols is treatment with DNase after the 0.2-μm-filtered (viral) fraction has been purified and enriched but before DNA extraction (11, 21, 30, 37). Under the assumption that most viral particles (virions) remain intact with their genomic contents protected at this stage, DNase treatment is meant to reduce the amount of extracellular and/or free, “relic” DNA (38) that may have been coenriched with the virions. The amount of relic DNA in a given soil sample presumably varies widely, depending on the soil, and the amount recovered in a given metagenomic or viromic library will also depend on the laboratory procedure(s) used to prepare the DNA (38–40). Estimates of relic DNA in soil vary (38, 39), but one study suggested that, on average, 40.7% of soil 16S rRNA gene amplicon sequences are relics (38). One meta-analysis of viromes (predominantly from freshwater, saline, and human gut environments, with none from soils) determined that a range of 0.2% to 40.3% of viromic reads were mapped to nonviral microbial genomes, suggesting the potential for substantial nonviral DNA contamination in some cases (41). However, the amount of free DNA contamination in soil viromes and the potential impact of this DNA on downstream analyses have yet to be thoroughly considered.

Although these previous results suggest that DNase treatment is an important step in the process of preparing a virome, the virions themselves can be compromised prior to DNA extraction such that DNase treatment of these compromised virions may remove the very viral genomic DNA that was meant to be enriched. Virions can be compromised naturally through degradation in the environment and potentially during sample collection, transportation, storage, and/or laboratory processing (42–44). In some cases, particularly if the virions were compromised after removal from the field, it may be desirable to recover DNA from these compromised virions. The successful enrichment of viral DNA via viromics without DNase treatment has been previously observed, for example, from a hypersaline lake and from soil (peat) samples stored frozen (19, 34). This suggests that in cases in which DNase treatment of a virome is not possible due to a loss of viral DNA, preparation of a virome that has not undergone DNase treatment may still be worthwhile. However, direct comparisons of DNase-treated and untreated viromes from the same samples have not been made in soil (or any other environment, to our knowledge), nor have these two types of viromes been placed in the context of recoverable viral sequences from total metagenomes.

Here, we sought to better understand the differences between soil viromes prepared with and without DNase treatment, in order to more thoroughly evaluate the utility of non-DNase-treated soil viromes (here, untreated viromes). Considering 15 viromes (7 DNase-treated [previously reported, with one having failed at the library construction step {30}] and 8 untreated [new in this study]) from 8 agricultural soil samples, this study compares the overall sequence complexities, assembly successes, proportions of recoverable viral contigs, percentages of viral reads, viral taxonomic diversities, and downstream ecological interpretations that would be derived from these two treatments. We hypothesized that treatment with DNase would increase the recovery of viral contigs by decreasing the overall sequence complexity and improving assembly and, therefore, that DNase treatment would be preferable, where possible. We also suspected that the overall patterns of viral community beta-diversity across samples would not be significantly influenced by DNase treatment and that untreated viromes would yield substantially more recognizable viral sequences than the total metagenomes that were previously sequenced from these same samples (30).

## RESULTS

### Comparison of metagenomic assembly success from DNase-treated and untreated viromes.

**FIG 1:**
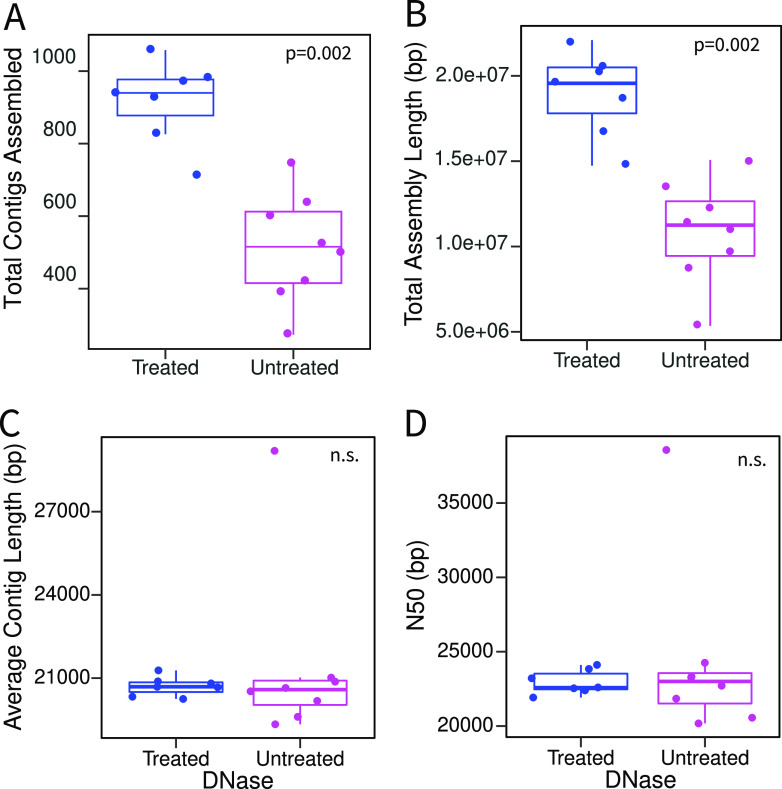
Assembly comparisons of DNase-treated and untreated viromes. Each point is one virome, with comparisons according to total contigs assembled (A), total assembly length (B), average contig length (C), and *N*_50_ (contig length where half the assembly length is represented in longer contigs and half is represented in shorter contigs) (D). Boxes show the interquartile ranges and median values. Whiskers extend to the furthest nonoutlying data point. *P* values show the significance of Kruskal-Wallis tests between DNase-treated (*n* = 7) and untreated (*n* = 8) samples. Insignificant results (*P* values of >0.05) are indicated as n.s. (nonsignificant).

We sampled eight agricultural plots that had been treated with four different biochar amendments (30) and generated two viromes (one treated with DNase and one untreated) from each sample. The DNase-treated viromes were part of a previous study (30), and the untreated viromes are new here. These 16 viromes were sequenced to a depth of 4 Gbp (range, 3.65 to 4.53 Gbp), apart from a single DNase-treated virome from which library construction failed, as previously described (see Table S2 in the supplemental material) (30). Despite equimolar DNA contributions to the sequenced pool of libraries, untreated samples recovered a greater number of sequencing reads than their DNase-treated counterparts (untreated median, 28,008,452; DNase-treated median, 26,847,586 [*P* = 0.02 by a Kruskal-Wallis test]) (Fig. S1). However, after quality filtering, there was no significant difference in the numbers of reads between treatment types (*P* = 0.08 by a Kruskal-Wallis test) (Fig. S1). Overall, DNase-treated viromes assembled into significantly more contigs (averages of 917 DNase-treated and 513 untreated contigs [*P* = 0.002 by a Kruskal-Wallis test]) and had a longer total assembly length than their paired untreated viromes (Fig. 1). However, the average contig lengths and *N*_50_s (i.e., the contig length where half the assembly length is represented in longer contigs and half is represented in shorter contigs) were statistically indistinguishable between the two treatments (Fig. 1; Table S3).

10.1128/mSystems.00614-21.1 FIG S1 Effect of DNase treatment on the number of reads generated per virome and the proportion of reads in each virome that mapped to each vOTU category. (A and B) Numbers of raw reads (A) and quality-filtered reads (B) generated per virome by DNase treatment. (C to E) Proportions of reads per virome that mapped to vOTUs assembled in solely DNase-untreated viromes (C), both DNase-treated and untreated viromes (D), and solely DNase-treated viromes (E). Boxes depict the interquartile ranges, with midlines representing the median values. Whiskers extend to the furthest nonoutlying data point. *P* values show the significance of Kruskal-Wallis tests between DNase-treated (*n* = 7) and untreated (*n* = 8) samples. Download FIG S1, PDF file, 0.02 MB. Copyright © 2021 Sorensen et al. 2021 Sorensen et al. https://creativecommons.org/licenses/by/4.0/ This content is distributed under the terms of the Creative Commons Attribution 4.0 International license.

10.1128/mSystems.00614-21.3 TABLE S2 Virome library and assembly metrics. Download Table S2, PDF file, 0.03 MB. Copyright © 2021 Sorensen et al. 2021 Sorensen et al. https://creativecommons.org/licenses/by/4.0/ This content is distributed under the terms of the Creative Commons Attribution 4.0 International license.

10.1128/mSystems.00614-21.4 TABLE S3 Kruskal-Wallis test of the effect of DNase treatment on different assembly metrics and viral and cellular organism-derived contents in viromes. Download Table S3, PDF file, 0.05 MB. Copyright © 2021 Sorensen et al. 2021 Sorensen et al. https://creativecommons.org/licenses/by/4.0/ This content is distributed under the terms of the Creative Commons Attribution 4.0 International license.

### Sequence complexity and proportion of cellular organism-derived reads in DNase-treated compared to untreated viromes.

**FIG 2:**
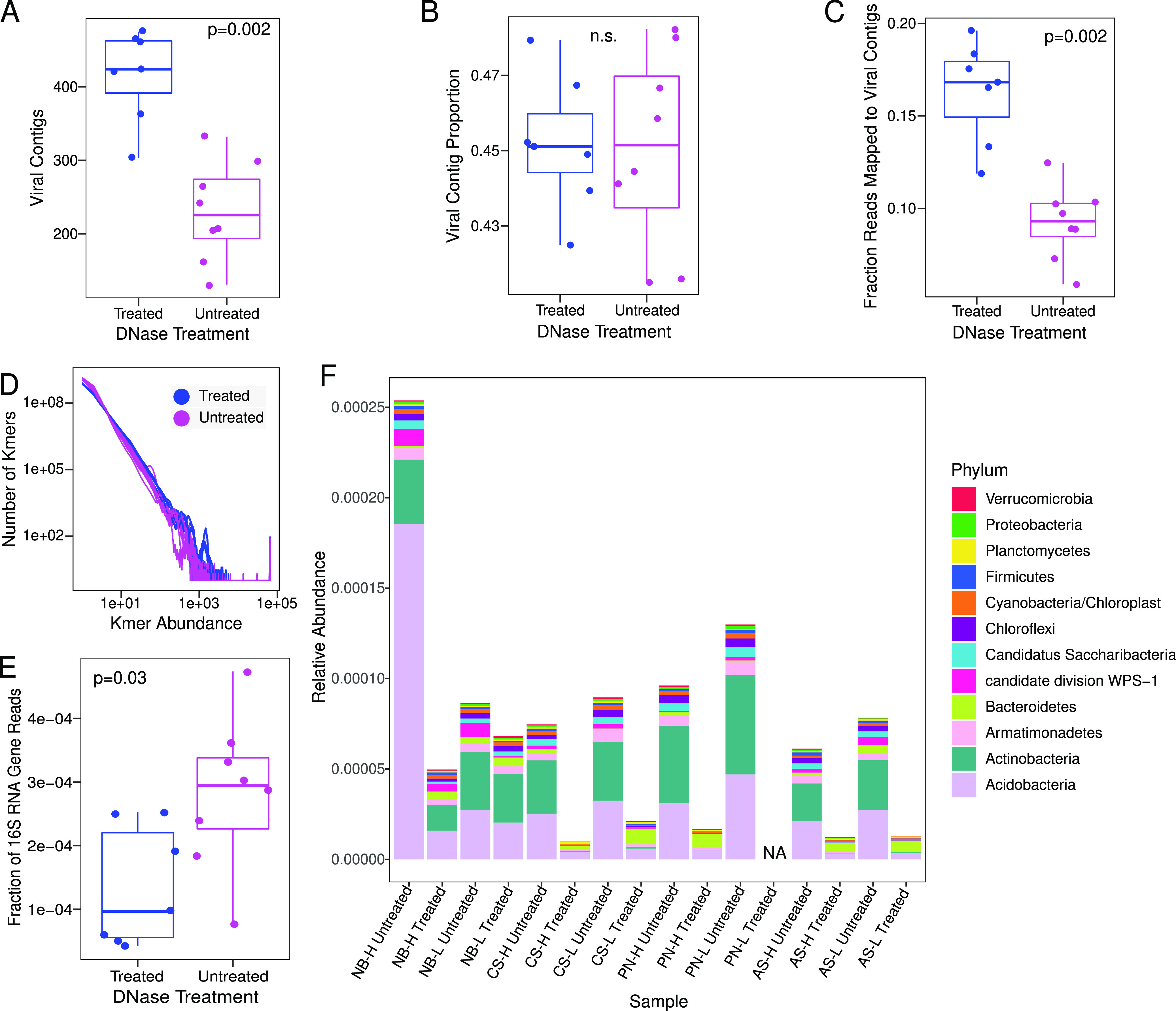
Differences in sequencing contents between DNase-treated and untreated viromes. (A and B) Number of VirSorter-identified viral contigs assembled per virome (A) and their proportion of the total number of contigs per virome (B). (C) Proportion of reads from each sample that mapped to VirSorter-identified viral contigs. (D) Frequency plot of k-mers showing k-mer abundance on the *x* axis and the number of k-mers with that abundance on the *y* axis. Each line is one virome. (E) Proportion of reads that contain partial 16S rRNA gene sequences as identified via SortMeRNA. (F) Relative abundances of the top 12 most abundant phyla according to partial 16S rRNA gene sequences. The *y* axis displays the number of reads containing 16S rRNA gene fragments from each of the top 12 phyla as a proportion of the total number of quality-trimmed reads in each virome. DNase-treated and untreated viromes from the same plot are placed next to each other for ease of comparison. NA, not applicable (no data). For all box plots (A to C and E), boxes show the interquartile ranges and median values, with whiskers extending to the furthest nonoutlying data point, and *P* values show the significance of Kruskal-Wallis tests between DNase-treated (*n* = 7) and untreated (*n* = 8) viromes. Insignificant results (*P* values of >0.05) are indicated as n.s. (not significant).

We suspected that the decreased sequence complexity in DNase-treated viromes contributed to the observed significant improvement in assembly, presumably due to the degradation of “free” DNA (e.g., from bacteria and archaea, as opposed to viruses). We tested this in two ways: first by comparing the k-mer complexity between the two approaches and second by comparing the 16S rRNA gene recovery rates. DNase-treated viromes tended to have more abundant k-mers and fewer singleton k-mers than their untreated counterparts (Fig. 2D), and DNase-treated viromes had significantly fewer total k-mers per sample (*P* = 0.002 by a Kruskal-Wallis test). We next asked whether the reduced complexity of the DNase-treated viromes could be attributable to a depletion of nonviral (e.g., bacterial and archaeal) DNA. Indeed, DNase-treated viromes had significantly fewer reads identifiable as 16S rRNA gene fragments by approximately 2-fold (on average, 0.013% for DNase-treated compared to 0.028% for untreated samples [*P* = 0.03 by a Kruskal-Wallis test]) (Fig. 2E; Table S3). Based on taxonomic classification of these 16S rRNA gene fragments, 9 of the 12 most abundant phyla across the data set had a significantly lower abundance in the DNase-treated viromes (Fig. 2F; Table S4), with *Acidobacteria*, *Actinobacteria*, and “*Candidatus* Saccharibacteria” showing the most significant differences between treatments. The phylum *Bacteroidetes* was the only phylum to increase in abundance in the DNase-treated viromes, and DNase treatment had no significant effect on *Planctomycetes* or *Verrucomicrobia* relative abundances.

10.1128/mSystems.00614-21.5 TABLE S4 Kruskal-Wallis test of the effects of DNase treatment on the recovery of the 12 most abundant bacterial phyla (in terms of total 16S rRNA gene read abundances across the data set). False discovery rate-adjusted *P* values are included to correct for the multiple phyla tested. Download Table S4, PDF file, 0.05 MB. Copyright © 2021 Sorensen et al. 2021 Sorensen et al. https://creativecommons.org/licenses/by/4.0/ This content is distributed under the terms of the Creative Commons Attribution 4.0 International license.

### Viral contig and viral population (vOTU) recovery from DNase-treated compared to untreated viromes.

We next wanted to assess whether treating viromes with DNase prior to DNA extraction had an influence on our ability to recover viral contigs and, subsequently, viral populations (viral operational taxonomic units [vOTUs]). We identified putative viral contigs from each single-sample assembly using VirSorter, retaining only viral contigs from the higher-confidence categories (categories 1, 2, 4, and 5) (10, 18, 25). Overall, DNase-treated viromes assembled significantly more putative viral contigs (median, 424; range, 303 to 475) than did untreated viromes (median, 226; range, 131 to 332 [*P* value of <0.01 by a Kruskal-Wallis test]) (Fig. 2A; Table S3).

**FIG 3:**
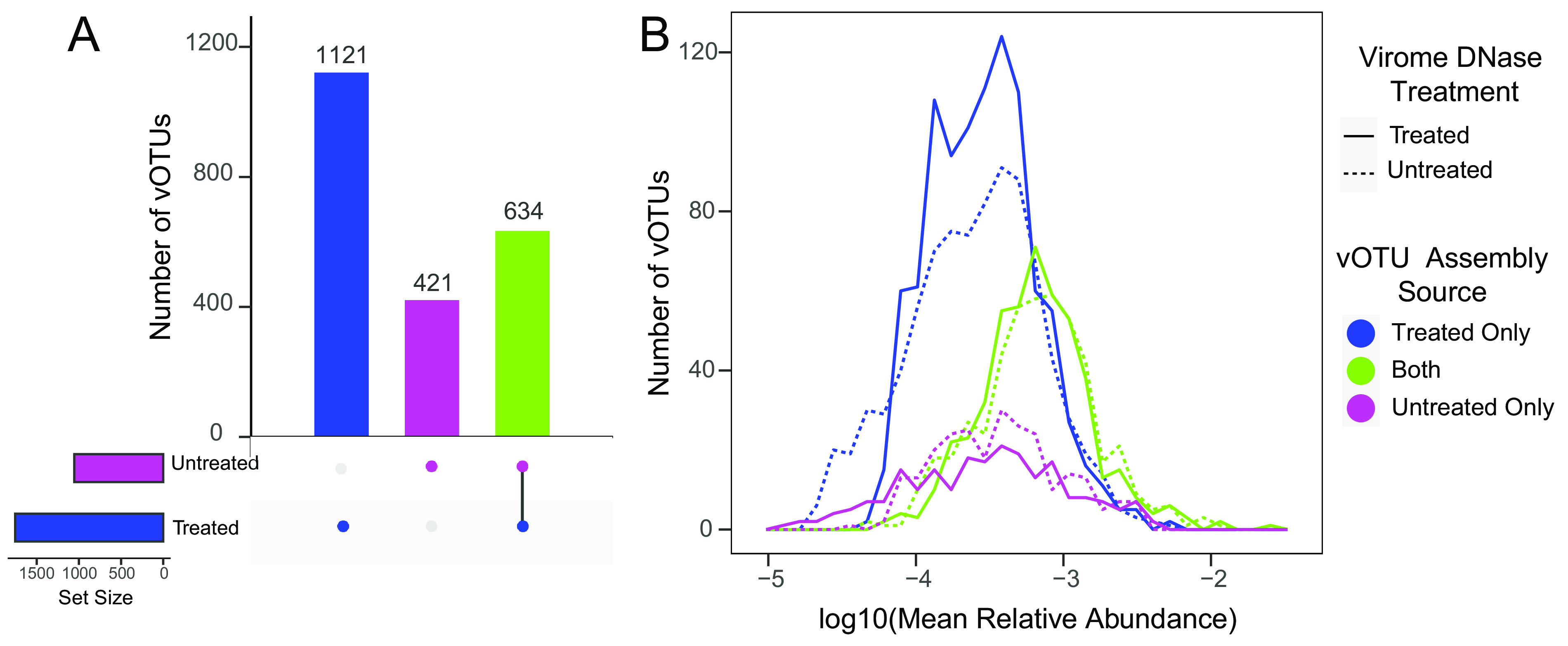
Numbers and relative abundances of vOTUs, according to the origin(s) of the assembled contigs contained within each vOTU. (A) Number of vOTUs that contained contigs clustered at 95% identity assembled from DNase-treated viromes only, untreated viromes only, or both types of viromes. (B) Distribution of vOTU mean relative abundances across viromes within each DNase treatment group, colored according to the assembly source(s) of viral contigs within each vOTU. Relative abundances are derived from read mapping such that vOTUs with contigs assembled solely from one treatment could have been detected in viromes from the other treatment via read recruitment.

Thus far, contigs from the same viral population could have been counted multiple times since each virome was assembled individually. In order to evaluate the recovery of unique viral populations (vOTUs), we clustered all of the putative viral contigs from both DNase-treated and untreated viromes at 95% nucleotide identity into vOTUs (36). We then categorized these vOTUs into three groups, according to the treatments from which their clustered contigs (representing the same viral “species”) were assembled, as follows: vOTUs containing contigs assembled solely from DNase-treated viromes (DNase vOTUs), assembled solely from DNase-untreated viromes (NoDNase vOTUs), or assembled in viromes from both treatments (shared vOTUs). In total, we identified 2,176 vOTUs, of which 1,121 were classified as DNase vOTUs, 421 were classified as NoDNase, and 634 were classified as shared (Fig. 3; Data Set S1). Thus, DNase treatment resulted in an ∼1.7-times-greater assembly of viral populations. However, of the 1,121 vOTUs that were assembled solely in DNase-treated viromes, 1,016 (90.6%) were detected in untreated viromes through read mapping, meaning that DNA from the vast majority of these vOTUs was present in the untreated viromes but did not sufficiently assemble.

10.1128/mSystems.00614-21.8 DATA SET S1 vOTUs identified in this study along with their length, viral cluster, taxonomy, and uniqueness based on assembled contigs. Download Data Set S1, XLSX file, 0.1 MB. Copyright © 2021 Sorensen et al. 2021 Sorensen et al. https://creativecommons.org/licenses/by/4.0/ This content is distributed under the terms of the Creative Commons Attribution 4.0 International license.

### Comparing the proportions of virus-derived reads in DNase-treated and untreated viromes.

With the set of 2,176 vOTUs as references for read mapping, we next sought to determine whether the smaller number of 16S rRNA gene reads was accompanied by an increase in viral reads in DNase-treated compared to untreated viromes. We mapped the quality-filtered reads from each sample to the dereplicated reference set of all vOTUs. Significantly higher numbers and fractions of reads from DNase-treated viromes mapped to vOTUs (on average, ∼3.2 million, or 17% of reads per sample) than in untreated viromes (on average, ∼1.9 million, or 9% of reads per sample) (Fig. 2C; Table S3). DNase treatment improved viral enrichment approximately 2-fold compared to untreated viromes.

### Patterns in the taxonomy and types of vOTUs assembled from DNase-treated and untreated viromes.

We next wanted to determine whether there were differences in the types of vOTUs recovered in DNase-treated compared to untreated viromes. We performed whole-genome, network-based clustering of predicted proteins using vConTACT2 (45) to cluster groups of vOTUs at approximately the genus level into viral clusters (VCs) (46). vConTACT2’s collection of viral genomes from the NCBI RefSeq database (ViralRefSeq-prokaryotes-v85) was included in this analysis for assigning taxonomy, as previously described (45). Of the 2,176 total vOTUs, 1,457 (67.0%) clustered into 744 VCs. A total of 599 VCs (80.5%) contained vOTUs assembled from both treatments, while 131 VCs (17.7%) exclusively contained vOTUs assembled from DNase-treated viromes, and 14 VCs (1.8%) exclusively contained vOTUs assembled from untreated viromes. Of the 744 total VCs, 295 were singletons containing only a single vOTU, of which the vast majority (240) were assembled from both treatments, while 44 singleton VCs were assembled solely in DNase-treated samples, and 11 singleton VCs were assembled only in DNase-untreated samples. Only 43 vOTUs (from 19 VCs) were assigned taxonomy, based on clustering in the same VC as a reference sequence, and these vOTUs accounted for 0.3% to 1.6% of the total viral community abundance (based on read mapping) in each virome. Of the 43 taxonomically classifiable vOTUs, 25 were assembled only in DNase-treated viromes, 11 were assembled only in DNase-untreated viromes, and 7 were assembled in both treatments. Considering these limited taxonomic assignments, DNase-treated and untreated viromes generally recovered the same taxonomic groups, namely, the *Caudovirales* families *Siphoviridae* (10 VCs), *Myoviridae* (5 VCs), and *Podoviridae* (4 VCs). We note that these results are based on the current taxonomies for the relevant reference sequences, but phage taxonomy is actively undergoing revision by the International Committee on Taxonomy of Viruses (ICTV), and these groups of *Caudovirales* have been recommended for removal as taxonomic groups (47).

### Recovery of relatively rare compared to abundant vOTUs by treatment.

We addressed relative-abundance patterns (for example, whether recovered vOTUs tended to be relatively abundant or rare) by comparing the proportions of reads recruited to vOTUs in the three different vOTU source categories (i.e., occurrence in assemblies within and/or across treatments, as described above) (Fig. S1). The shared vOTUs (those assembled in at least one virome from both treatments) recruited on average 57.7% and 59.5% of mapped reads from DNase-treated and untreated viromes, respectively, despite these shared vOTUs accounting for only approximately 29% of the total vOTUs (634/2,176) (Fig. S1). While vOTUs uniquely assembled from DNase-treated viromes accounted for 52% of all vOTUs (1,121/2,176), they recruited only an average of 32.5% of mapped reads from DNase-treated viromes and a similar but slightly lower percentage of mapped reads from untreated viromes (28.9%) (Fig. S1). These results led us to suspect that the vOTUs uniquely assembled in DNase-treated viromes tended to be relatively rare (in low abundance) compared to the vOTUs assembled in both treatments. To address this, we constructed frequency plots of the mean relative abundances of vOTUs by category (treatment specific or shared) (Fig. 3). In both DNase-treated and untreated viromes, the distribution of treatment-specific vOTU abundances was shifted to the left (indicating lower abundances), compared to the abundances of shared vOTUs (*P* < 0.001 by a Kruskal-Wallis test). In untreated viromes, vOTUs assembled only from untreated viromes had mean relative abundances similar to those of vOTUs assembled only from DNase-treated viromes. In contrast, in DNase-treated viromes, vOTUs assembled only from DNase-treated viromes had significantly higher relative abundances than vOTUs assembled only from untreated viromes (*P* < 0.001 by a Kruskal-Wallis test). In short, while there were some vOTUs that were both uniquely assembled in one treatment and in high abundance in one or both treatments, the vast majority of the treatment-specific vOTUs were present in low relative abundances compared to those that were assembled in both DNase-treated and untreated viromes.

### Ecological inferences from DNase-treated compared to untreated viromes.

**FIG 4:**
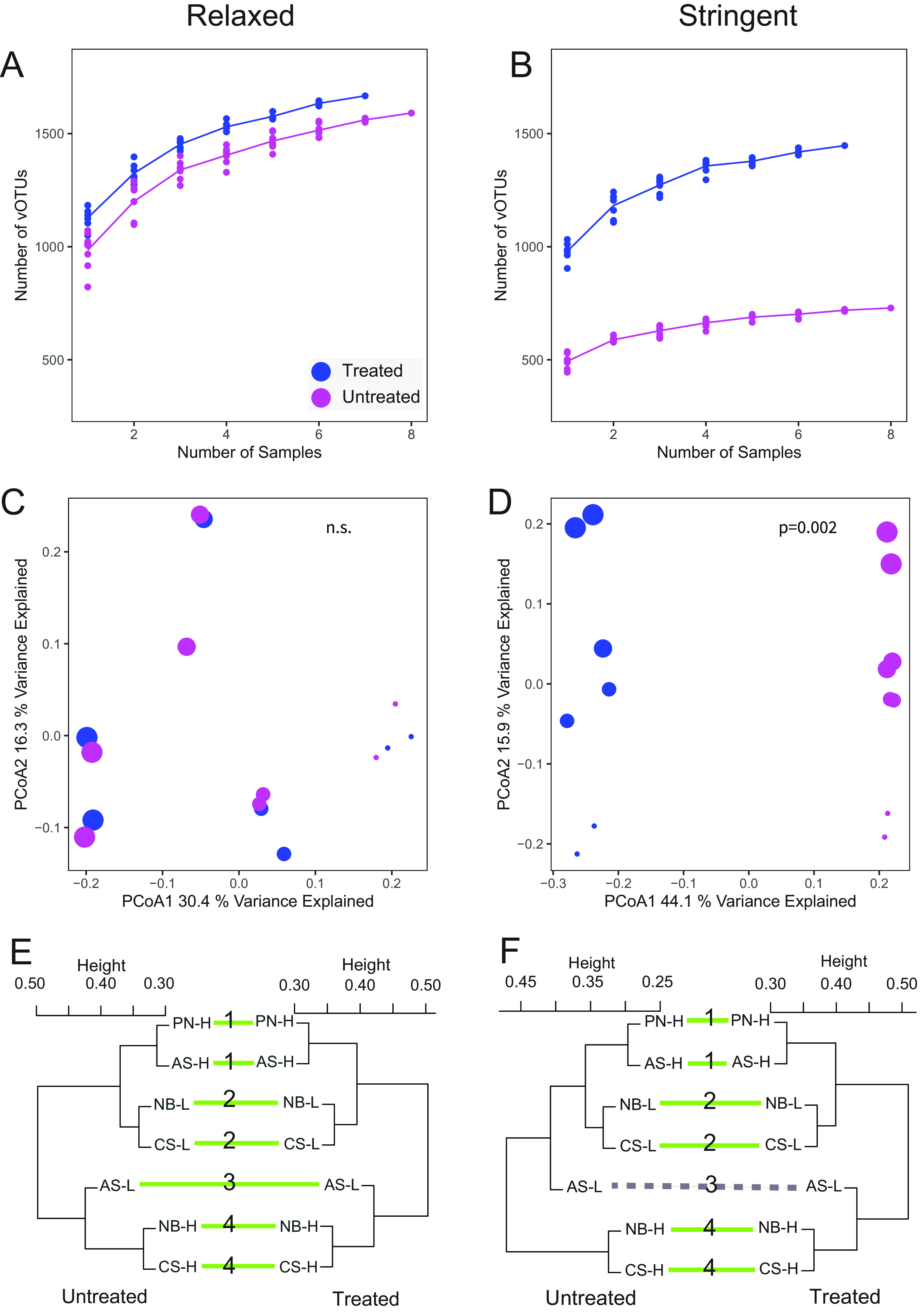
Comparisons of ecological properties across DNase treatments and vOTU detection criteria. Results from the “relaxed” vOTU detection criteria (left) and the “stringent” vOTU detection criteria (right) are shown (relaxed detection allowed read mapping to all vOTUs in the data set, and stringent detection allowed mapping only to vOTUs derived from the same virome treatment group). (A and B) Accumulation curves showing the total number of vOTUs detected within a DNase treatment group with different numbers of samples. (C and D) Principal-coordinate analyses (PCoAs) of Bray-Curtis dissimilarities (each point is one virome), labeled by DNase treatment (color) and location along the E-W axis of the sampled field (shape size, where the largest symbols correspond to locations farthest East, with decreasing size along the E-W axis). *P* values show the significance of DNase treatment on community structure using PerMANOVA. (E and F) Tanglegrams, each linking two sets of hierarchical clusters of viral community composition (one per DNase treatment group). Green lines connect samples with congruent clustering between the two treatment groups, and dashed lines connect samples with discongruent clustering. Numbers in the middle of each tanglegram correspond to the plot’s location along the E-W axis of the sample field. Dendrograms were created using complete linkage clustering with Bray-Curtis dissimilarities. In panels E and F, the untreated virome from plot PN-L was removed, as it did not have a paired DNase-treated virome. As a result, there was only one virome per treatment (from plot AS-L) at that particular E-W location within the field; all other E-W locations were represented by two viromes per treatment.

In order to better understand how DNase treatment, or a lack thereof, might influence downstream ecological interpretations of soil viromic data, we applied and compared two different sets of vOTU detection criteria. For both analyses, we followed the same established best practices for considering a vOTU to be “detected” in a given sample (36). The first set of detection criteria, which we refer to as “relaxed,” considers data from reads mapped to all 2,176 reference vOTUs (i.e., vOTUs assembled from any virome in this study). The second set of criteria, referred to as “stringent,” removed from consideration reads that mapped to vOTUs that were assembled only from the other treatment group. This stringent set of criteria was meant to mimic a data set in which only one treatment had been performed (DNase treated or untreated), as would be expected for most viromic studies. DNase-treated viromes had significantly higher perceived richness (alpha-diversity) than their untreated counterparts for both the relaxed (on average, 1,128 versus 985 vOTUs [*P* = 0.003 by a Kruskal-Wallis test]) (Fig. 4A and B; Table S5) and stringent (on average, 980 versus 494 vOTUs per untreated virome [*P* = 0.001 by a Kruskal-Wallis test]) (Table S5) criteria. While both DNase-treated and untreated viromes had lower observed richness using the stringent criteria, the untreated samples showed a greater decrease (approximately 2-fold) in richness between the relaxed and stringent criteria.

10.1128/mSystems.00614-21.6 TABLE S5 Richness values (numbers of vOTUs detected) for each virome based on relaxed and stringent detection criteria. Download Table S5, PDF file, 0.03 MB. Copyright © 2021 Sorensen et al. 2021 Sorensen et al. https://creativecommons.org/licenses/by/4.0/ This content is distributed under the terms of the Creative Commons Attribution 4.0 International license.

We also wanted to determine whether DNase treatment affected analyses of viral community structure. Using both the relaxed and stringent criteria for vOTU detection, we calculated pairwise Bray-Curtis dissimilarities between viromes. With the relaxed criteria, there was no significant difference in viral community composition attributable to DNase treatment (Fig. 4C) (*P* = 0.952 by permutational multivariate analysis of variance [PerMANOVA]), but the application of the stringent criteria resulted in a significant effect of DNase treatment (Fig. 4D) (*P* = 0.002). We also wanted to assess whether one set of viromes exhibited a larger amount of variation than the other. When using the relaxed vOTU criteria, the beta-dispersion (i.e., the breadth of beta-diversity within a group) of the DNase-treated and untreated viromes was statistically indistinguishable (*P* = 0.430 for homogeneity of multivariate dispersions). When applying the stringent vOTU detection criteria, the DNase-treated viromes trended toward showing greater beta-dispersion, but the difference was not statistically significant (*P* = 0.075 for homogeneity of multivariate dispersions).

Finally, we previously observed a strong East-to-West (E-W) gradient effect on the viral community composition in these agricultural fields, using DNase-treated viromes only (30). Under the assumption that this gradient effect was real, we assessed our ability to detect this effect in both the DNase-treated and untreated viromes. We observed a significant East-West structuring of the viral community composition in both sets of viromes using both relaxed and stringent criteria (Fig. 4C and D; Table S6). We further confirmed the robustness of viral community compositional patterns to DNase treatment by testing for correlations between the Bray-Curtis community dissimilarity matrices derived from DNase-treated compared to untreated viromes using Mantel tests. The observed beta-diversity patterns (i.e., how samples were grouped according to viral community composition) were highly correlated between DNase-treated and untreated viromes, according to both stringent and relaxed vOTU detection criteria (Mantel *R* values of 0.87 for relaxed criteria and 0.83 for stringent criteria [both *P* = 0.002]). This result was further reinforced in a tanglegram, which showed highly similar hierarchical clusterings of samples according to viral community composition between the two virome treatments, independent of vOTU detection criteria (Fig. 4E and F). A single sample (AS-L) clustered differently in tanglegrams derived from DNase-treated compared to untreated data when using the stringent vOTU detection criteria only. This was the only sample that lacked a paired sample from the same East-West position in the field, owing to the necessary removal of the single successful virome from that plot (plot PN-L) in this analysis, because its matched DNase-treated virome failed at the library construction step. Otherwise, each pair of samples from the same field column grouped together in all four hierarchical clusters (DNase treated versus untreated and relaxed versus stringent vOTU detection criteria).

10.1128/mSystems.00614-21.7 TABLE S6 PerMANOVA results showing the effects of DNase treatment and location along the East-to-West axis of the sampled field (E-W gradient) on the viral community structure under two different vOTU detection criteria (relaxed and stringent [see Materials and Methods]). Bray-Curtis dissimilarities were used for these analyses. Download Table S6, PDF file, 0.02 MB. Copyright © 2021 Sorensen et al. 2021 Sorensen et al. https://creativecommons.org/licenses/by/4.0/ This content is distributed under the terms of the Creative Commons Attribution 4.0 International license.

### Comparison of viral recovery from untreated viromes and total soil metagenomes.

In most metrics that we have compared to this point, DNase-treated viromes have outperformed untreated viromes, but we wanted to know the extent to which untreated viromes could still improve viral sequence recovery and reduce bacterial and archaeal DNA contents in viromes compared to total soil metagenomes. We previously analyzed total soil metagenomes from these same samples (30), which showed an average of 2.2% viral contig content (compared to 45% for untreated viromes in this study, an ∼20-fold improvement), 0.04% 16S rRNA gene reads (compared to 0.02% for untreated viromes here), and an average of 0.04% of reads mapping to vOTUs (compared to 9.2% for untreated viromes here, an ∼225-fold improvement). Furthermore, the ecological patterns observed in this study were robust to different DNase treatments (Fig. 4), and we wanted to know the extent to which mining total metagenomes for viral signatures would yield the same patterns. For example, a highly significant effect of spatial structuring (E-W gradient effect) on viral community composition was observed for untreated viromes here, even with the stringent detection criteria (*P* = 0.003 by PerMANOVA), and we wanted to know the extent to which this pattern could also have been recovered from the total soil metagenomes. While this result was reproduced with viral communities recovered from the total soil metagenomes, the significance was borderline (*P* = 0.045 by PerMANOVA).

## DISCUSSION

### DNase treatment of viromes reduced contamination and sequence complexity, consistent with the removal of free DNA.

We have shown that DNase treatment of viromes significantly reduced sequence complexity and decreased the amount of contaminating cell-derived DNA (measured as 16S rRNA gene fragments) by about 2-fold. Sequence complexity has long been a challenge for assembling environmental metagenomes and can result in high fragmentation of genomes from low-abundance species (48, 49). Thus, we suspect that the observed decrease in sequence complexity in DNase-treated viromes was responsible for the larger, more contiguous assemblies from DNase-treated viromes, and it is reasonable to assume that this reduction in sequence complexity resulted from free (relic) DNA depletion as a result of successful DNase treatment.

Relic DNA (sometimes called environmental DNA [eDNA] or free DNA) is not contained within a viable cell or virion and has been shown to artificially increase the observed richness of microbial communities in some soils (38, 39), presumably by allowing the detection of locally dead or extinct microbial taxa (38). Studies have also suggested that the presence of relic DNA can obscure or minimize patterns in beta-diversity (38, 50, 51), but here, we observed that both DNase-treated and untreated viromes produced viral communities with highly correlated beta-diversity patterns (Fig. 4). Although there was a single sample that clustered differently in DNase-treated compared to untreated viromes when using the stringent vOTU detection criteria, we attribute this difference predominantly to the lack of a successful replicate matching sample in the same column of the field rather than differences in relic DNA compositions between treatments.

### Viromics without DNase treatment might be particularly useful for samples stored frozen.

The laboratory protocol for generating viromes requires equipment that is unlikely to be available or practical to run in the field, precluding immediate processing of samples collected from distant field sites (19, 34). Even samples from nearby sites may need to be stored temporarily, as a relatively small number of samples can be processed for viromics at a time (6 to 12 per ∼2 days in our laboratory, but this will depend on the available equipment and personnel). Frozen storage can preserve *in situ* community composition (52–55), and ideally, virions would be frozen in a cryoprotectant or a similar substance to preserve their integrity, but the compatibility of cryoprotectants with various viromics protocols is not well known. Thus, in some cases, direct freezing of samples may be necessary (19). We have previously shown that freezing can prohibit the use of DNase on aquatic viromes, resulting in viral DNA yields below detection limits (34), and anecdotally, we have seen similar results from soils stored frozen (data not shown).

Encouragingly, work from our group has shown that viromes prepared without DNase treatment (untreated viromes) from frozen peat soils can still substantially improve vOTU recovery, compared to total metagenomes (19). Similarly, hypersaline lake water stored frozen yielded predominantly viral sequences in viromes that did not undergo DNase treatment (34). In combination with the complete depletion of DNA after DNase treatment in these hypersaline lake samples, it is reasonable to suspect that some virions became compromised by freezing such that DNase treatment removed valuable viral genomic DNA contained in degraded virions that may have been intact in the field. Studies in pure culture support virion degradation through freezing; for example, coliphages from wastewater showed decreased viability after prolonged storage in frozen wastewater, and Bacillus subtilis bacteriophage viability decreased by multiple logs after only 2 h of frozen storage with no cryoprotectant (43, 44).

Here, to ensure that we could obtain sufficient DNA for sequencing from both treatments for a direct comparison, we compared fresh soil samples (stored at 4°C and processed within 1 week of sample collection) with and without DNase treatment. While results from this and previous studies converge to suggest that skipping DNase treatment is likely to be a good option for viromics from samples stored frozen, future comparisons would benefit from the inclusion of a combination of samples processed fresh and after frozen storage.

### Recommendations for future viral ecology studies.

We have shown here that DNase treatment produced better assemblies, more viral contigs, fewer 16S rRNA gene reads (indicative of bacterial and archaeal DNA), and more viral reads than not treating samples with DNase. However, both kinds of viromes substantially outperformed total soil metagenomes (30) in these metrics. Together, these results suggest that soil viromics with DNase treatment is the best approach for interrogating soil viral ecology, where possible, but soil virome preparation without DNase treatment can be better than total soil metagenomics when DNase treatment is not an option. Previous work suggests that these results may be generalizable to viromics in other ecosystems as well (34), but to our knowledge, direct comparisons of these approaches have not been made in other ecosystems. The decision of what approach to take is inherently dependent on the questions being asked, along with the logistics of sample collection, storage (and possibly shipment), and performing laboratory sample processing. While we expect shotgun metagenomic approaches to consistently underrepresent viral diversity across most soils, it is possible that the effects of DNase treatment on viromes could vary across different soil ecosystems and physicochemical conditions. Based on the results from the samples tested here, where possible, we recommend processing soil viromes fresh (without frozen storage) and soon (within ∼1 to 5 days) after sampling, as prolonged storage even at 4°C can lead to viral degradation (44). Furthermore, we also recommend the inclusion of DNase treatment after virion purification and before virion lysis, particularly for fresh soils.

However, even without DNase, soil viromes substantially enrich for viral sequences in comparison to total soil metagenomes. Across multiple studies including fresh, frozen, agricultural, and peat soil samples in various combinations (19, 30; this study), viromics (with or without DNase) seems to outperform total metagenomics for soil viral community investigations. Still, only a tiny fraction of soil types and a few combinations of laboratory procedures have been attempted, so assessing the broad generalizability of the observed trends will require expanding our investigations across diverse terrestrial and other ecosystems. Thus far, the extra effort required to purify virions from soil prior to DNA extraction seems to be worthwhile, even without DNase treatment.

## MATERIALS AND METHODS

### Sample collection and soil processing.

Our sampling design and soil collection process have been described previously (30). Briefly, eight agricultural tomato plots near the University of California, Davis (UC Davis), campus (38°32′08″N, 121°46′22″W) were sampled on 23 April 2018. Each of the plots had been treated with one of four biochar amendments (650°C pyrolyzed pine feedstock, 650°C pyrolyzed coconut shell, 800°C pyrolyzed almond shell, or no biochar control) on 8 November 2017 as part of an ongoing study to investigate the impact of biochar treatment on agricultural production (see Table S1 in the supplemental material). Tomato seedlings had not yet been planted at the time of sampling (the field was fallow). The top 30 cm of soil was collected using a 2.5-cm-diameter probe, and a total of 8 probe cores per plot were combined into a single sterile bag per sample and transported on ice to the laboratory, where each sample was sieved through 8-mm mesh.

10.1128/mSystems.00614-21.2 TABLE S1 Relevant metadata for viromes under BioProject accession number PRJNA646773 (additional metadata reported previously by C. Santos-Medellin, L. A. Zinke, A. M. ter Horst, D. L. Gelardi, et al. [ISME J 15:1956–1970, 2021, https://doi.org/10.1038/s41396-021-00897-y]). All treated viromes were first reported by Santos-Medellin et al., and untreated viromes are first reported in this study. Download Table S1, PDF file, 0.03 MB. Copyright © 2021 Sorensen et al. 2021 Sorensen et al. https://creativecommons.org/licenses/by/4.0/ This content is distributed under the terms of the Creative Commons Attribution 4.0 International license.

### Viral purification and DNA extraction for viromics.

The eight DNase-treated viromes were prepared as previously described (30), and in the current study, the same soil samples were also prepared without DNase treatment, for a total of 16 samples. The laboratory processing steps for all samples were the same up to the DNase treatment step. Briefly, viromes were generated for each sample from 50 g of fresh soil separated into two 50-ml conical tubes, according to a previously described protocol (11), with slight modifications (30). To each of the two tubes per sample, 37.5 ml of 0.02-μm-filtered AKC′ extraction buffer (10% phosphate-buffered saline [PBS], 10 g/liter potassium citrate, 1.44 g/liter Na_2_HPO_4_, 0.24 g/liter KH_2_PO_4_, 36.97 g/liter MgSO_4_) (37) was added. Tubes were briefly vortexed to homogenize the soil slurry and then shaken at 400 rpm for 15 min on an orbital shaker. Subsequently, each tube was vortexed for an additional 3 min before undergoing centrifugation at 4,700 × *g* for 15 min to pellet the soil. The two supernatants from the same sample were then filtered through a 0.22-μm polyethersulfone filter to remove most cells and combined into a 70-ml polycarbonate ultracentrifuge tube, which was centrifuged for 3 h at 4°C at 32,000 × *g* to pellet viral particles. Taking care not to disturb the pellet, the supernatant was discarded, and the viral pellet was resuspended in 200 μl of ultrapure water. The eight untreated samples (no DNase treatment) proceeded directly to DNA extraction at this point. To the eight samples designated for DNase treatment, 30 U of RQ1 RNase-free DNase and 30 μl of 10× DNase buffer (Promega Corp., Madison, WI, USA) were added, and samples were incubated at room temperature for 2 h before stopping the reaction with 30 μl of the DNase stop solution (Promega Corp., Madison, WI, USA), as previously described (30). The eight DNase-treated samples underwent DNA extraction at this point. For both DNase-treated and untreated viromes, DNA was extracted using the DNeasy PowerSoil kit (Qiagen, Hilden, Germany) according to the manufacturer’s instructions, with slight modifications, as previously described (30).

### Library construction and sequencing.

Libraries were constructed and sequenced by the DNA Technologies and Expression Analysis Core at the UC Davis Genome Center. The DNA Hyper Prep library kit (Kapa Biosystems-Roche, Basel, Switzerland) was used for all libraries. A single lane of an Illumina HiSeq 4000 paired-end 150-bp sequencing platform was used to generate all of the sequencing data, with a targeted sequencing depth of 4 Gbp per sample.

### Sequence processing, assembly, and identification of viral contigs.

All viromes were bioinformatically processed from raw sequencing data (i.e., those that were previously reported were reprocessed here). Sequencing reads were quality trimmed and primers were removed using Trimmomatic (56). MEGAHIT was used with the “meta” preset to individually assemble each virome, using the paired quality-trimmed reads and a minimum contig length of 10 kbp (57). Analyses of overall assembly statistics (Fig. 1) were performed on these data. Putative viral contigs were then identified using VirSorter (v1.0.6) in decontamination mode (–virome), retaining any contigs that were assigned to higher-confidence categories (1, 2, 4, or 5), in accordance with established recommendations (10, 18, 25, 28). Analyses considering viral contigs not yet dereplicated into populations (Fig. 2A to C) were performed on these data.

### k-mer analyses of virome sequence complexity.

k-mer counting was performed using the khmer software package version 2.1.1 (58). Reads were first k-mer error trimmed using the command “trim-low-abund.py” before k-mers of size 31 were counted in each virome using the script “load-into-counting.py.”

### Taxonomic identification of bacterial and archaeal 16S rRNA gene contents in viromes.

SortMeRNA was used with its internal SILVA bacterial and archaeal 16S rRNA gene databases (version V119) to identify partial 16S rRNA gene sequences present in the reads from each virome (59, 60). Reads found to contain partial 16S rRNA gene sequences were then classified using the Ribosomal Database Project classifier trained with the RDP training set and a confidence cutoff of 0.8 (61). Classifications were collapsed at the phylum level to create a phylum-by-sample table in order to investigate changes in the relative abundances of phyla across DNase treatments.

### Viral population (vOTU) identification, read mapping, and vOTU detection criteria for ecological analyses.

VirSorter-identified viral contigs (described above) were dereplicated through clustering, using the “psi-cd-hit.pl” command of CDHIT (62) with a minimum alignment length equal to 85% of the smaller contig and a minimum percent identity equal to 95%, in accordance with best practices for identifying viral populations (vOTUs) (37). The resulting representative seed contig sequences from each cluster were then used as our set (“database”) of vOTUs for further analysis. vOTU representative seed sequences were annotated using prodigal (63) and then grouped into viral clusters (VCs) and taxonomically identified using vConTACT2 (v0.9.19) with its “ProkaryoticViralRefSeq85-Merged” database (45) and the default settings.

In order to perform community ecological analyses, the relative abundances of each vOTU in each sample were assessed by read mapping to the reference database of vOTUs. Specifically, quality-trimmed reads were mapped to the database of vOTUs at a minimum identity of 90% using BBMap (64). The resulting SAM files were then converted into sorted and indexed BAM files using SAMtools (65). The trimmed pileup coverage and read count abundance of each vOTU were calculated using BamM parse to generate tables of vOTU abundances (average coverage depth) in each sample (66). We used bedtools to calculate the per-base coverage for each vOTU in each sample, requiring that >75% of the vOTU contig length be covered by at least one read for detection in a given virome (also known as “breadth”) (67, 68). The vOTU coverage tables generated to this point were considered in analyses with “relaxed” detection criteria, meaning that reads that mapped to vOTUs assembled from any sample were included. For analyses using “stringent” detection criteria, we also required that for a given vOTU to be considered detected in a virome, an assembled contig from that same virome and/or another virome within the same DNase treatment group had to be in the same ≥95% nucleotide identity vOTU cluster. In other words, that same vOTU (viral “species”) must have been assembled from a virome in the same treatment group, mimicking conditions under which only that treatment had been performed and thus only vOTUs from that treatment would be in the reference database for read mapping. The resulting vOTU coverage tables were used for downstream ecological and statistical analyses.

### Ecological and statistical analyses.

After generating the vOTU tables, all ecological and statistical analyses were performed in R (69). The vegan package was used to calculate Bray-Curtis dissimilarities (function vegdist) using vOTU relative abundances, perform PerMANOVA (function adonis), and correlate matrices (Mantel tests, function mantel) (70). In order to perform a nonparametric test for the differences between two unevenly sized groups of nonnormally distributed data, Kruskal.test from the stats package was used to perform the Kruskal-Wallis rank sum test. Box plots were constructed using ggplot2, and tanglegrams were constructed using the dendextend package (71, 72). For both Mantel tests and tanglegram analyses comparing DNase-treated and untreated viromes, the untreated virome from plot PN-L was dropped from the analysis because its paired DNase-treated virome failed at the library construction step.

### Data availability.

All viromes analyzed and presented in the current study have been deposited in the NCBI SRA under BioProject accession number PRJNA646773.

10.1128/mSystems.00614-21.9 DATA SET S2 vOTU coverage tables using relaxed vOTU detection criteria. Download Data Set S2, XLSX file, 0.2 MB. Copyright © 2021 Sorensen et al. 2021 Sorensen et al. https://creativecommons.org/licenses/by/4.0/ This content is distributed under the terms of the Creative Commons Attribution 4.0 International license.

10.1128/mSystems.00614-21.10 DATA SET S3 vOTU coverage tables using stringent vOTU detection criteria. Download Data Set S3, XLSX file, 0.2 MB. Copyright © 2021 Sorensen et al. 2021 Sorensen et al. https://creativecommons.org/licenses/by/4.0/ This content is distributed under the terms of the Creative Commons Attribution 4.0 International license.
