# Supplementary material for: DNase Treatment Improves Viral Enrichment in Agricultural Soil Viromes
Source: mSystems. 2021 Sep 7;6(5):e00614-21. doi: 10.1128/mSystems.00614-21 (PMC8547471; doi:10.1128/mSystems.00614-21)
Supplement: TABLE S2 [file msystems.00614-21-st002.pdf]

Table S2 Virome library and assembly metrics

Alternate ViromeID	Raw Reads	Quality Reads	Assembled Contigs	Assembly Length(bp)	Average Contig Length(bp)	N50(bp)
NB-H_Treated	27221284	19301736	929	18815260	20253	21914
NB-L_Treated	27180638	19700594	713	14737975	20670	22395
CS-H_Treated	26177978	19198294	970	20640671	21279	24107
CS-L_Treated	26847586	19616206	940	19564995	20814	23218
PN-H_Treated	25463842	18763090	826	16791656	20329	22581
AS-H_Treated	27356524	20303230	1058	22095334	20884	23830
AS-L_Treated	24373292	17460364	984	20357992	20689	22546
NB-H_Untreated	28931300	21417332	392	11444745	29196	38579
NB-L_Untreated	30182260	22144462	641	13475530	21023	24267
CS-H_Untreated	27715654	19845492	603	12378175	20528	22725
CS-L_Untreated	28719338	21244602	423	8731895	20643	23325
PN-H_Untreated	27195428	19558874	501	9686598	19335	20559
PN-L_Untreated	28301250	20773902	273	5352575	19607	20181
AS-H_Untreated	26326880	18440362	747	15072298	20177	21830
AS-L_Untreated	27271098	19579214	530	11062615	20873	23273
