# Supplementary material for: DNase Treatment Improves Viral Enrichment in Agricultural Soil Viromes
Source: mSystems. 2021 Sep 7;6(5):e00614-21. doi: 10.1128/mSystems.00614-21 (PMC8547471; doi:10.1128/mSystems.00614-21)
Supplement: TABLE S3 [file msystems.00614-21-st003.pdf]

Table S3 Kruskal Wallis test of the effect of DNase treatment on different assembly metrics and viral and cellular organism-derived content in viromes.

**Metric**	**KW Chi-Squared**	**P value**
Contig Count	9.76	0.002
Assembly Length	9.76	0.002
Average Contig Length	0.48	0.487
N50	0.00	1.000
16S rRNA gene reads	4.83	0.028
Viral contigs (total)	9.76	0.002
Viral contigs (proportion of total contigs)	0	1
Total reads mapped to viral contigs	10.5	0.001
Proportion of reads mapped to viral contigs	9.76	0.002
