# Supplementary material for: DNase Treatment Improves Viral Enrichment in Agricultural Soil Viromes
Source: mSystems. 2021 Sep 7;6(5):e00614-21. doi: 10.1128/mSystems.00614-21 (PMC8547471; doi:10.1128/mSystems.00614-21)
Supplement: TABLE S4 [file msystems.00614-21-st004.pdf]

Table S4 Kruskal Wallis test of the effects of DNase treatment on recovery of the 12 most abundant bacterial.

Phylum	KW Chi-Squared	P value	fdr adjusted p value
Acidobacteria	10.5	0.001	0.014
Actinobacteria	9.76339	0.002	0.020
Armatimonadetes	9.05357	0.003	0.023
Bacteroidetes	6.48214	0.011	0.044
candidate division WPS-1	7.13579	0.008	0.038
Candidatus Saccharibacteria	9.76339	0.002	0.020
Chloroflexi	8.37054	0.004	0.027
Cyanobacteria/Chloroplast	7.71429	0.005	0.033
Firmicutes	6.48214	0.011	0.044
Planctomycetes	1.92857	0.165	0.330
Proteobacteria	9.06977	0.003	0.023
Verrucomicrobia	0.12054	0.728	0.728
