# Supplementary material for: DNase Treatment Improves Viral Enrichment in Agricultural Soil Viromes
Source: mSystems. 2021 Sep 7;6(5):e00614-21. doi: 10.1128/mSystems.00614-21 (PMC8547471; doi:10.1128/mSystems.00614-21)
Supplement: TABLE S5 [file msystems.00614-21-st005.pdf]

Table S5 Richness values (number of vOTUs detected) for each virome based on relaxed and stringent detection criteria

Virome_ID	Relaxed Richness	Stringent Richness
NB-H_Treated	1104	963
NB-H_Untreated	916	459
NB-L_Treated	1050	904
NB-L_Untreated	1061	530
CS-H_Treated	1156	1011
CS-H_Untreated	1006	490
CS-L_Treated	1183	1032
CS-L_Untreated	1012	501
PN-H_Treated	1124	976
PN-H_Untreated	967	488
PN-L_Untreated	822	445
AS-H_Treated	1139	986
AS-H_Untreated	1071	536
AS-L_Treated	1139	986
AS-L_Untreated	1022	499
