# Supplementary material for: DNase Treatment Improves Viral Enrichment in Agricultural Soil Viromes
Source: mSystems. 2021 Sep 7;6(5):e00614-21. doi: 10.1128/mSystems.00614-21 (PMC8547471; doi:10.1128/mSystems.00614-21)
Supplement: TABLE S6 [file msystems.00614-21-st006.pdf]

Table S6 PerMANOVA results showing the effects of DNase treatment and location along the East to West axis of the sampled field (E-W Gradient) on viral community structure under two different vOTU detection criteria.

Viromes Analyzed	vOTU Detection Criteria	Independent Variable	Pseudo F	R^2	p value
All viromes (15)	Relaxed	DNase treatment	0.415	0.031	0.952
All viromes (15)	Stringent	DNase treatment	10.2	0.44	0.002
All DNase treated (7)	Relaxed	E-W Gradient	2.663	0.348	0.004
All DNase untreated (8)	Relaxed	E-W Gradient	2.776	0.316	0.003
All DNase treated (7)	Stringent	E-W Gradient	2.582	0.341	0.002
All DNase untreated (8)	Stringent	E-W Gradient	2.511	0.295	0.003
