# Supplementary material for: DNase Treatment Improves Viral Enrichment in Agricultural Soil Viromes
Source: mSystems. 2021 Sep 7;6(5):e00614-21. doi: 10.1128/mSystems.00614-21 (PMC8547471; doi:10.1128/mSystems.00614-21)
Supplement: TABLE S1 [file msystems.00614-21-st001.pdf]

Table S1 Relevant metadata for viromes in BioProject accession PRJNA646773 (additional metadata appear in Santos-Medellin et al. 2021, ISMEJ)

ViromeID	SRA	PlotID	DNaseTreatment	BiocharTreatment	Column
B0H_VFD_S52	SRR8487010	NB-H	Treated	None	4
B0L_VFD_S51	SRR8487011	NB-L	Treated	None	2
B2R2H_VFD_S54	SRR8487017	CS-H	Treated	Coconut	4
B2R2L_VFD_S53	SRR8487016	CS-L	Treated	Coconut	2
B3R2H_VFD_S55	SRR8487020	PN-H	Treated	Pine	1
B8R2H_VFD_S57	SRR8487022	AS-H	Treated	Almond	1
B8R2L_VFD_S56	SRR8487019	AS-L	Treated	Almond	3
B0H_VFN_S44	SRR14509072	NB-H	Untreated	None	4
B0L_VFN_S43	SRR14509071	NB-L	Untreated	None	2
B2R2H_VFN_S46	SRR14509070	CS-H	Untreated	Coconut	4
B2R2L_VFN_S45	SRR14509069	CS-L	Untreated	Coconut	2
B3R2H_VFN_S48	SRR14509068	PN-H	Untreated	Pine	1
B3R2L_VFN_S47	SRR14509067	PN-L	Untreated	Pine	3
B8R2H_VFN_S50	SRR14509066	AS-H	Untreated	Almond	1
B8R2L_VFN_S49	SRR14509065	AS-L	Untreated	Almond	3
